# Penile fracture: A case report from Nepal

**DOI:** 10.1002/ccr3.4786

**Published:** 2021-09-05

**Authors:** Alok Atreya, Suman Baral

**Affiliations:** ^1^ Department of Forensic Medicine Lumbini Medical College Palpa Nepal; ^2^ Department of Surgery Lumbini Medical College Palpa Nepal

**Keywords:** injuries, penile fracture, sexual activity, surgical repair

## Abstract

Penile fractures are clinically diagnosed with eggplant‐like deformity of the penis (aubergine sign). Surgical repair immediately following the penile fracture is the standard of care, which usually heals with minimal or no complications.

## Graphical abstract

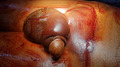

Penile fractures are readily diagnosed clinically with eggplant‐like deformity of the penis (aubergine sign). Patients might present late to the clinician's attention owing to fear and embarrassment. Surgical repair immediately following the penile fracture is the standard of care, which usually heals with minimal or no complications.

A 26‐year‐old man came to ED at early morning hour with a complaint of pain in the penis for 4 h. The patient stated that he felt severe pain and a snapping sound during the sexual act with his wife with woman on top position. On examination, the penis was circumcised, swelled, and discolored blue. There was a deviation of the penis toward the left (Figure 1). There was no voiding difficulty or history of hematuria.

**FIGURE 1:**
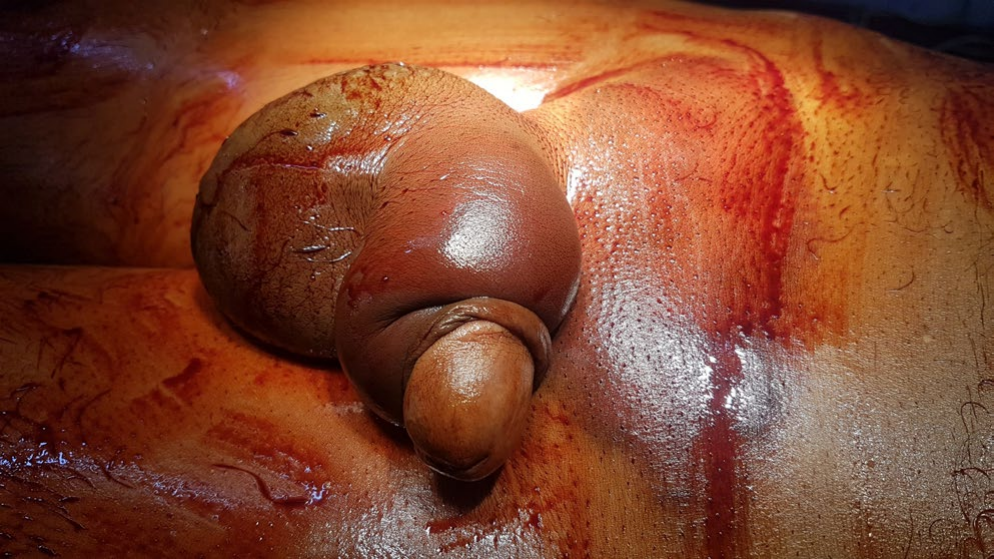
Eggplant‐like deformity of the penis (aubergine sign)

Catheterization was done with 16 Fr Foley's catheter. Inj. Cefazoline 2 g was given intravenously as prophylaxis. A sub‐coronal circumferential incision was given on the penile shaft. The penis was degloved, the hematoma was evacuated, and the injury was explored (Figure 2). The injury on tunica albuginea was repaired with a 3‐0 vicryl continuous inverting suture. The Foley catheter was removed on the first post‐operative day. The patient had an uneventful recovery and was discharged on the fifth day of admission. The patient was prescribed oral antibiotics, analgesics, and sedatives. The patient was advised to abstain from sexual activity for three months as per the local practice.

**FIGURE 2:**
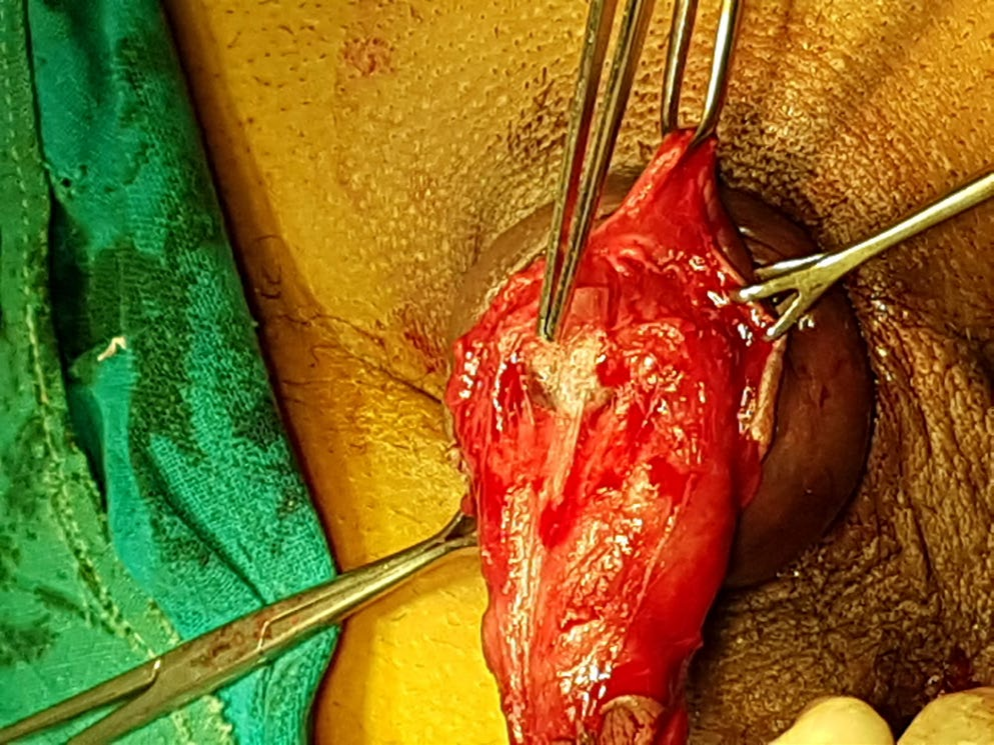
Exposure of the injured tunica albuginea during surgery

Fracture of the penis commonly occurs during violent sexual activity with reported incidence of 1 in 175,000 in US male population,^1^ is still under reported in developing countries. Surgical repair immediately following the penile fracture is the standard of care, which usually heals without any complications.^2^ Penile fractures can be easily diagnosed clinically as a radiological confirmation is not mandatory. If there is no bleeding from meatus or a history of hematuria, the urethra is intact. In complicated cases with a urethral tear, it is advocated to refer the patient to a higher center with the Urology unit.

## CONFLICT OF INTEREST

None declared.

## AUTHOR CONTRIBUTION

AA wrote the original draft. SB involved in patient care and revised the manuscript.

## ETHICAL APPROVAL

Ethics approval is not applicable.

## CONSENT

Published with written consent of the patient.

## Data Availability

Data sharing is not applicable to this article as no datasets were generated or analyzed during the current study.
